# Effects of Running Speeds and Exhaustion on Iliotibial Band Strain during Running

**DOI:** 10.3390/bioengineering10040417

**Published:** 2023-03-26

**Authors:** Shanefei Chen, Yan Wang, Fangbo Bing, Ming Zhang

**Affiliations:** 1 Department of Biomedical Engineering, Faculty of Engineering, The Hong Kong Polytechnic University, Hong Kong 999077, China; 2 Hong Kong Polytechnic University Shenzhen Research Institute, Shenzhen 518057, China; 3 Research Institute for Sports Science and Technology, The Hong Kong Polytechnic University, Hong Kong 999077, China

**Keywords:** biomechanics, exhaustion, running speed, iliotibial band

## Abstract

Background: Iliotibial band syndrome (ITBS) is one of the most prevalent overuse injuries in runners. The strain rate in the iliotibial band (ITB) has been theorized to be the primary causative factor in the development of ITBS. Running speed and exhaustion might lead to an alteration in the biomechanics that influence the strain rate in the iliotibial band. Objectives: To identify how exhaustion states and running speeds affect the ITB strain and strain rate. Methods: A total of 26 healthy runners (including 16 males and 10 females) ran at a normal preferred speed and a fast speed. Then, participants performed a 30 min exhaustive treadmill run at a self-selected speed. Afterward, participants were required to run at similar speeds to those of the pre-exhaustion state. Results: Both the exhaustion and running speeds were revealed to have significant influences on the ITB strain rate. After exhaustion, an increase of approximately 3% in the ITB strain rate was observed for both the normal speed (*p* = 0.001) and the fast speed (*p* = 0.008). Additionally, a rapid increase in the running speed could lead to an increase in the ITB strain rate for both the pre- (9.71%, *p* = 0.000) and post-exhaustion (9.87%, *p* = 0.000) states. Conclusions: It should be noted that an exhaustion state could lead to an increase in the ITB strain rate. In addition, a rapid increase in running speed might cause a higher ITB strain rate, which is proposed to be the primary cause of ITBS. The risk of injury should also be considered due to the rapid increase in the training load involved. Running at a normal speed in a non-exhaustive state might be beneficial for the prevention and treatment of ITBS.

## 1. Introduction

Iliotibial band syndrome (ITBS) is one of the most prevalent overuse injuries in runners, which leads to lateral knee pain [1]. The iliotibial band (ITB) originates from the iliac crest and distally inserts into Gerdy’s tubercle with attachments at the lateral femoral epicondyle [2]. In a previous study, the ITB was found to store about 5% of the elastic energy during a normal-paced run using a three-dimensional musculoskeletal model of the lower limb [3]. ITBS accounts for over 12% of all running-related injuries [4], which might lead to psychological disorders. Furthermore, ITBS was also found to be the most prevalent injury in soccer and basketball players [5]. One predominant theory of ITBS pathomechanics proposed that the ITB compresses against the lateral femoral epicondyle medially due to the increase in the ITB tension when the knee flexion angle is over 30° [6]. The strain rate of the ITB was presented as a major factor in the development of ITBS via a musculoskeletal model incorporated into the ITB in OpenSim [7]. Some previous studies also discovered factors that influence ITB strain. The increases in the hip adduction and knee internal rotation angles were found to be related to the causes of a higher ITB strain rate [7,8]. Sinclair et al. [9] examined different movements, including running, cutting, and hopping, which indicated that running and cutting resulted in a higher amount of strain than hopping. Additionally, a narrower step width was revealed to increase the ITB strain rate significantly when compared to a preferable condition [10]. It was also proposed that females exhibit a higher peak strain and a higher strain rate due to an increase in the hip internal rotation angle compared to males during overground running [11]. Due to the complex anatomical attachments and insertions, the ITB strain and strain rate might be changed by the biomechanics of the knee and hip joints.

Running speed is one of the main influential factors in gait. Compensatory strategies might be adopted when runners increase their speeds. One dominant strategy is for runners to shift to increase their stride cadence and push on the ground with a higher frequency, which consequently applies more force on the hip joint as the running speed increases [12]. The increase in their cadence was revealed to lead to a decrease in the hip adduction angle and hip adductor moment [13]. Faster running speeds were found to increase the knee joint loads per stride during overground running [14]. Due to the influences of the running speed on the biomechanics of the lower limb, the potential contributions of the running speed to the strain and strain rate of the ITB still remain unknown.

Prolonged, exhaustive running has significant effects on the biomechanics of the lower limb joints, which are essential intrinsic factors of running-related injuries [15,16]. A significant increase in segment coordination variability was found to coincide with the prolongation of the running mileage [17]. The moments of the hip joints were observed to increase significantly at the initial contact after a 5 km treadmill run [15]. Therefore, the strain and strain rate of the ITB might be altered by an exhaustive run. Although Miller et al. [18] investigated the mechanics of the ITB during an exhaustive run, some limitations were observed. These running trials were performed on a treadmill, which might have led to different kinematics and kinetics of the lower limb joints compared with overground running. Additionally, participants wore their own shoes during the running, despite another study suggesting that footwear affects the ITB strain significantly [19].

It has also been implied that excessive mileage is associated with the development of ITBS [14]. An alternative injury factor was determined to be running speed. However, few studies to date have examined the interactive influences of exhaustion states and running speeds on the strain and strain rate of the ITB, which have been proposed to be the essential factors in the development of ITBS. Thus, in this study, both the running speed and the exhaustion states were examined to alter the kinematics and kinetics of the lower limb. The purpose of the current study was to identify the influences of the running speed, before and after an exhaustive run, on the strain and strain rate of the ITB. We hypothesized that both the exhaustion states and increase in running speed could cause an increase in the ITB strain rate.

## 2. Methodology

### 2.1. Participants

Twenty-five participants (including sixteen males and ten females) were recruited for this study. Details about the participants are listed in Table 1. All participants were healthy and free of lower limb injuries in the preceding year. The age range of the participants was between 20 and 30 years old, and their BMI was between 20 and 24. All of the participants had a habit of running. Informed written consent documents were signed by all participants before the experiment. The Human Subjects Ethics Sub-Committee of the Hong Kong Polytechnic University (number: HSEARS20150121003) approved this study.

### 2.2. Experiments

**Figure 1:**
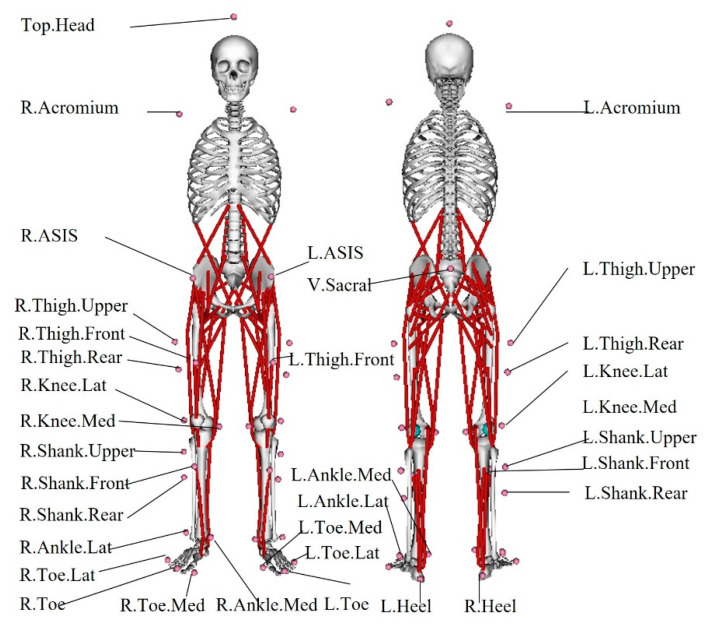
The musculoskeletal model with the marker set.

An eight-camera motion-capture system (Vicon, Oxford Metrics Ltd., Oxford, UK) was used to collect the motion data at a sampling frequency of 250 Hz. One force plate (OR6, AMTI, Watertown, MA, USA) was used to record the ground reaction force at a sample frequency rate of 1000 Hz, which recorded simultaneously with the Vicon system. The Vicon system was calibrated prior to each session to ensure that the image error was below 0.2 mm. Before the formal tests, a total of 34 markers were attached to the feet, legs, and torso of each participant. Reflective markers were placed on the anatomical landmarks: the forehead; acromioclavicular joints; posterior and anterior iliac spines; three skin-mounted in a triangle on the thigh; lateral and medial femoral epicondyles; three skin-mounted in a triangle on the shank; lateral and medial malleoli; the heads of the first and fifth metatarsals; and the tip of the toes. It was ensured that each segment possessed three markers that were used to track 3-dimensional motion, as shown in Figure 1.

Each participant wore lab-provided sports shoes (ARHQ025-4, Li-Ning Inc., Beijing, China) of appropriate size. Participants were encouraged to become familiar with the shoes and warm up. Data of a static trial were collected for each participant standing on the force plate, which were used to scale the generic musculoskeletal model. Then, dynamic trials were performed using the normal running speed and a faster speed that was just over 10% of each participant’s normal speed. The two speed conditions were confirmed by the participants, with the speeds in a random order. A successful trial was confirmed by stepping on the force plate with the entire right foot. In addition, the speed tolerance of each trial of the same speed needed to be within ±5% to conduct the analysis. Five successful trials were collected for each condition. Afterward, each participant was asked to perform a treadmill run at their self-selected speed for 30 min, with each of them ultimately reaching an exhaustion state. Then, participants were asked to perform the dynamic overground running trials again. All participants performed running trials with a preferred speed and a fast speed during their pre- and post-treadmill runs, running at a self-selected speed for 30 min. The normal speed and the fast speed were maintained at approximately the same level. Real-time feedback on speed was provided for participants to confirm that the speed conditions were maintained similarly before and after the treadmill run. Before the formal tests, participants were required to conduct at least ten warm-up overground running trials to resume their natural gait. Notably, participants were not allowed to rest in order to maintain the exhaustive state. Their running speeds were confirmed using the average speed of the anterior–posterior speeds of the sacral marker, which could provide feedback immediately after each trial [20]. Details of the running are shown in Table 1. Marker trajectories and ground reaction forces were recorded simultaneously for all trials.

### 2.3. MSK Model

The musculoskeletal model of the ITB was established in OpenSim [7]. The model includes the feet, shanks, patellae, thighs, pelvis, torso, and head. The hip joint processes six degrees of freedom that enable rotation and translation. The knee joint was modified to be a three-degrees-of-freedom joint from a single-degree-of-freedom joint. The ITB was presented as an elastic structure running from the iliac crest to the Gerdy’s tubercle, following the anatomical pathway of the TFL in the model [7]. The rest length of the ITB was defined as the ITB length in the static calibration model scaled by the static trial. The strain and strain rates were calculated using the scaled musculoskeletal model, which was driven by the dynamic data of each participant. Then, the peak ITB strain and strain rate were extracted to perform the analysis. The length of the ITB in the stance phase during running could be obtained from the model. The strain of the ITB at each time step was calculated via the formula [7]:

$$Strain=\frac{L_{i}-L}{L}$$

where $L_{i}$ is the ITB length at the time i during the stance phase, and L is the rest length. The strain rate of the ITB was calculated via the first central difference method for each time step, which is shown as follows:

$$Strain\;rate=\frac{Strain_{i+1}-Strain_{i-1}}{Time_{i+1}-Time_{i-1}}$$

### 2.4. Data Analysis

SPSS software (Version 16.0; SPSS Inc., Chicago, IL, USA) was used to perform all of the statistical analyses. The significance level for all tests was set at 0.05. The mean variables were calculated by averaging the five successful trials, allowing for statistical analysis. The kinematics and kinetics of the knee and hip joints were obtained to perform the analysis. The strain and strain rate were calculated to conduct statistical analyses under different conditions. All variables were established to be normally distributed using the Shapiro–Wilk test. Independent paired *t*-tests were used to evaluate the group differences for different speed levels. The differences in the kinematics and kinetics of the hip and knee joints, and also in the strain and strain rate of the ITB between exhaustion (before and after) and speed levels, were examined using a two-way repeated-measures analysis of variance (ANOVA). Mauchly’s test was adopted to examine the sphericity assumption of the repeated-measures ANOVA. The results revealed that all of the variables passed the assumption. The moments were normalized according to the mass of the participants. Post-hoc tests were performed for significant ANOVA interactions using *t*-tests with Bonferroni corrections. The post-hoc tests with the adjustment of the Bonferroni method confirmed the presence of significant differences.

## 3. Results

No significant interaction was identified for all variables of interest between the exhaustion states and running speeds. The kinematic parameters of the knee and hip joints were influenced by both speed and exhaustion states, as shown in Table 2. The hip flexion angle showed a significant increase of about 5° as the running speed improved, and about 3° when comparing the results from before and after the exhaustive run at the same running speed. The hip adduction angle revealed a slight increase (*p* = 0.002) at the fast speed after the exhaustive run but showed no significant difference when the running speed changed. On the contrary, the hip internal rotation angle decreased significantly (*p* = 0.001) after an exhaustive run when the running speed increased. As for the knee joint, when the speed increased, the knee flexion angle revealed a significant increase under the same exhaustive state. Additionally, the angle was found to increase significantly after exhaustive running when the running speed remained similar.

The kinetics of the knee and hip joints were also influenced by the exhaustion states and running speeds, as shown in Table 3. The peak moment of the hip extension tended to increase significantly when the running speed improved under both the pre- and post-exhaustion states. However, the exhaustion states revealed no significant effects on the hip extension moment. The peak moment of the hip internal rotation also showed a significant increase (*p* = 0.007) during the exhaustive state when the running speed increased. The peak moment of the knee extension revealed a significant increase (*p* = 0.002) at the normal speed after exhaustive running. However, the running speed showed no significant effects on the knee extension moment.

Peak ITB strains were similar when the running speed increased, as shown in Table 4. Additionally, exhaustion states showed no effects on the ITB strain. Instead, both the exhaustion states and running speeds revealed significant influences on the ITB strain rate. As the running speed increased, the ITB strain rate became significantly larger, with an approximate increase of 10% for both the pre- and post-exhaustion states. Furthermore, as the running speed remained similar, the ITB strain rate was found to have a significant increase of about 4% after an exhaustive run.

## 4. Discussion

The current study aimed to explore how exhaustion states (before and after an exhaustive run) and running speeds altered the behaviors of the ITB due to the changes in the kinematics and kinetics of knee and hip joints. The flexion angles of both the knee and hip joints revealed significant increases when the running speed improved. In addition, the angles increased significantly when participants experienced an exhaustive run. The peak angle of the hip adduction also increased significantly when comparing pre- and post-exhaustion states at a fast speed. The greater hip adduction was associated with the increase in the ITB strain [21]. The running speed exhibited a negative relationship with the hip internal rotation. The peak angle of the hip internal rotation revealed a significant decrease when the running speed improved after an exhaustive run. The kinetics of the hip and knee joints also displayed alterations due to different exhaustion states and running speeds. The peak moment of the hip extension increased significantly due to the increase in the running speed. Additionally, the peak hip internal rotation moment revealed an increase when the running speed improved after an exhaustive run. Both the exhaustion states and the running speeds made contributions to the changes in the ITB strain rate. However, no significant interactions were found between the exhaustion states and the speeds for the ITB strain and strain rate. The ITB could be regarded as a passive tissue without voluntary contractions, which could contribute to the absorption of tension during running [22]. Greater ITB tension resulted in an increase in the compression between the ITB and lateral femoral epicondyle, indicating a predisposition to ITBS.

The peak of the ITB strain remained similar when the running speed increased. Furthermore, it showed no significant changes after an exhaustive run. However, the peak of the ITB strain rate revealed a significant increase alongside the increase in running speeds for both the pre- and post-exhaustion states. Additionally, the ITB strain rate also showed a significant increase after an exhaustive run. The increase in the ITB strain rate has been proposed to be a major causative factor in the development of ITBS [7]. The increase in the ITB strain rate was proposed to lead to higher tension in the ITB [7], which potentially increased the compression force between the ITB and the epicondyle. The strain rate increased by approximately 3% for both the normal speed and fast speed before and after the exhaustive run. The ITB strain rate was found to have a similar increase in similar running speeds after an exhaustive run, which revealed that the exhaustion states and the running speeds had no significant interactive influences on the ITB strain rate. Furthermore, the strain rate increased by approximately 10% when comparing the fast speed level to the normal speed level under the same exhaustion state. The mechanical behaviors of the ITB might be affected by the alteration in the proximal factors at the hip joint and the distal factors at the knee joint, which is due to the multiple anatomical insertions [11]. The gluteus maximus muscle partly inserts into the ITB, and thus plays an important role in its mechanical functions [23]. The gluteus maximus muscle contributes to the rotation of the hip joint, which causes tension to the ITB [21]. As the running speed increases, a much larger muscle force might be transformed from the gluteus maximus muscle, which leads to the increase in the ITB tension. Therefore, an increase in the running speed was proposed to influence the ITB strain rate in a more severe way when the running speed is faster.

The current study found that the peak hip adduction angle showed a significant increase when participants ran at a fast speed before and after an exhaustive run, which is in agreement with previous findings [24]. The peak hip adduction angle also showed an increase when participants ran at a normal speed after exhaustion, though not significantly. However, the increase in the angle was proposed to be related to the development of ITBS [25]. The ITB acts to resist hip adduction due to the demand for lateral hip stabilization [26], which might lead to an increase in ITB tension due to the increased hip adduction angle. As the hip adduction increases in the exhaustion state, a higher hip adduction moment might occur due to the higher eccentric demand of the gluteal musculature [8]. Though no significant effects were displayed in regard to the peak moment of the hip abduction, an increase was still observed. The increase in the hip adduction was related to weaker hip strength, which is an essential factor in limiting and controlling the peak of the hip adduction angle during the stance phase of running [27]. The exhaustive run was proposed to induce weak hip strength that would lead to an increase in the peak hip adduction angle. For both the normal speed and fast speed, the peak of the ITB strain rate increased by approximately 4% after an exhaustive run. This might be induced by the increase in the hip adduction angle. Consequently, exhaustion states were determined to be an influential factor in the ITB strain rate.

With respect to the influences of the running speeds, the peak angle of the hip internal rotation tended to decrease as the running speeds increased when participants were in the post-exhaustion state. During the pre-exhaustion state, a small decrease was also observed in the hip internal rotation angle. There were no significant differences in the similar speeds between pre- and post-exhaustion states, which was in agreement with a previous study [28]. The increase in the peak hip internal rotation angle was shown to reduce the ITB strain rate [11]. However, as the running speed increased, the peak angle of the hip internal rotation decreased, which might lead to an increase in the ITB strain rate. Repetitive loading onto the lower limb joints might lead to overuse injuries [29]. The decrease in the range of hip internal rotation could lead toward the increasing chance of predisposing to ITBS. Running speeds also caused a significant increase in the peak knee flexion angle. A greater knee flexion angle was proposed to increase the elongation of the ITB, which might lead to a larger ITB strain rate [11]. The results might imply that a rapid increase in training intensity, especially with an increase in running speed, could cause an increased risk of ITB-related injuries, especially ITBS.

In this study, a few limitations should be considered. Participants in this study were healthy runners, meaning the results might not be generalizable to injured populations. Additionally, gender differences were not a part of the investigation in the current study. The musculoskeletal model is not able to incorporate the complex anatomical structures of the ITB. Validation of the ITB model would be very difficult with non-invasive measures. The ITB was simulated to be completely passive and its length was represented by the length between the connected markers [11]. All of the parameters analyzed in this study were calculated using a musculoskeletal model. However, the peak strains (9–10%) were within the normal in vivo limits of elastic structures (5–12%) [30], and were lower than the failure strain of about 13% reported in a cadaver test from a previous study [31]. As the ITB is simulated as passive soft tissue in the model, influential factors from the in-series musculature of the ITB are not discussed in the current study. Although the running speeds were controlled to remain the same before and after the exhaustion state through real-time feedback during tests, small differences still exist within the same running speed levels. Additionally, we discussed the effects of the normal speed condition and fast speed condition on the strain and strain rate of the ITB before and after exhaustion. The model could not calculate the compression force between the ITB and the femoral epicondyle, which is the pain area for ITBS. Future investigations should incorporate the complex anatomy structures of the ITB with in-series muscles to examine the impact factors of the muscles and the compression forces. Finally, all of the participants recruited in the current study were healthy runners, so the results may not propagate to injured populations.

## 5. Conclusions

In this study, a musculoskeletal model was used to examine the influences of the exhaustion states and running speeds on the ITB strain and strain rate. The hip adduction angle revealed an increase that corresponded with the exhaustion state. The peak of the hip internal rotation angle decreased due to the increase in running speed. Both the exhaustion states and running speeds showed a significant effect on the ITB strain rate. The results imply that a rapid increase in the training intensity might predispose healthy runners to sustain higher ITB strain rates, which might lead to injuries, such as ITBS.

## Figures and Tables

**Table 1:** Information regarding the participants and experiments.

Participant Characteristics		Value
Mass/kg		64.73 (±11.65)
Height/cm		170.90 (±8.38)
Pre-30 min run	Normal speed/(m/s)	3.33 (±0.36)
	Fast speed/(m/s)	3.90 (±0.41)
Post-30 min run	Normal speed/(m/s)	3.32 (±0.36)
	Fast speed/(m/s)	3.93 (±0.38)

**Table 2:** Peak joint angles for different speeds for pre- and post-conditions.

Joint Angles/°	Before Exhaustion (Mean ± SD)		After Exhaustion (Mean ± SD)	
	Normal Speed	Fast Speed	Normal Speed	Fast Speed
Hip flexion	29.70 (±4.00)	34.96 (±5.19) ^b^	32.40 (±4.85) ^a^	37.86 (±4.55) ^a,b^
Hip adduction	13.89 (±2.85)	14.22 (±3.21)	15.15 (±2.99)	15.48 (±2.82) ^a^
Hip internal rotation	7.90 (±6.56)	6.97 (±5.92)	8.01 (±5.82)	5.63 (±6.22) ^b^
Knee flexion	44.59 (±3.55)	46.81 (±3.86) ^b^	46.94 (±3.69) ^a^	49.09 (±3.43) ^a,b^
Knee adduction	5.92 (±3.28)	6.44 (±3.46)	6.26 (±3.91)	6.71 (±4.11)
Knee internal rotation	7.67 (±5.29)	7.99 (±5.84)	8.92 (±6.22)	8.722 (±6.70)

^a^: significant difference in exhaustion states from the matching speed condition (*p* < 0.05). ^b^: significant difference in speed levels from the matching pre-exhaustion condition (*p* < 0.05).

**Table 3:** Peak joint moments for different speeds for pre- and post-conditions.

Moment (Nm/kg)	Before Exhaustion (Mean ± SD)		After Exhaustion (Mean ± SD)	
	Normal Speed	Fast Speed	Normal Speed	Fast Speed
Hip extension	2.33 (±0.62)	2.77 (±0.51) ^b^	2.42 (±0.47)	2.87 (±0.60) ^b^
Hip abduction	1.18 (±0.39)	1.42 (±0.72)	1.32 (±0.42)	1.47 (±0.47)
Hip internal rotation	0.26 (±0.11)	0.32 (±0.14)	0.31 (±0.10)	0.36 (±0.13) ^b^
Knee extension	1.73 (±0.32)	1.75 (±0.30)	1.87 (±0.36) ^a^	1.86 (±0.32)
Knee adduction	0.66 (±0.30)	0.76 (±0.48)	0.73 (±0.31)	0.79 (±0.38)
Knee internal rotation	0.25 (±0.15)	0.31 (±0.26)	0.28 (±0.19)	0.32 (±0.20)

^a^: significant difference from the matching speed condition (*p* < 0.05). ^b^: significant difference from the matching pre-exhaustion condition (*p* < 0.05).

**Table 4:** Peak ITB strain and strain rate for different speeds for pre- and post-conditions.

Variables	Before Exhaustion (Mean ± SD)		After Exhaustion (Mean ± SD)	
	Normal Speed	Fast Speed	Normal Speed	Fast Speed
ITB strain/%	9.54 (±0.59)	9.56 (±0.66)	9.63 (±0.70)	9.53 (±0.80)
ITB strain rate/(%/s)	35.05 (±7.79)	44.76 (±8.86) ^b^	38.57 (±9.04) ^a^	48.44 (±10.90) ^a,b^

^a^: significant difference in exhaustion states from the matching speed condition (*p* < 0.05). ^b^: significant difference in speed levels from the matching pre-exhaustion condition (*p* < 0.05).

## Data Availability

Not applicable.
